# Nanocellulose reinforced lightweight composites produced from cotton waste via integrated nanofibrillation and compounding

**DOI:** 10.1038/s41598-023-29335-z

**Published:** 2023-02-07

**Authors:** Dan Liang, Wangcheng Liu, Tuhua Zhong, Hang Liu, Renuka Dhandapani, Hui Li, Jinwu Wang, Michael Wolcott

**Affiliations:** 1 Apparel, Merchandising, Design and Textiles, Washington State University, Pullman, WA 99164 USA; 2 Composite Materials and Engineering Center, Washington State University, Pullman, WA 99164 USA; 3 International Centre for Bamboo and Rattan, Beijing, 100102 China; 4 Cotton Incorporated, Cary, NC 27513 USA; 5 Forest Products Laboratory, U.S. Forest Service, Madison, WI 53726 USA

**Keywords:** Synthesis and processing, Nanoscale materials

## Abstract

Cotton is a natural fiber containing more than 95% of cellulose. With worldwide cotton consumption continuously increasing, the amount of cotton waste generated is enormous. Most of the cotton waste ends up in landfill or incinerators, resulting in a huge waste of this excellent natural resource. In this project, cotton waste was recycled to produce polypropylene nanocomposites. Instead of using the traditional two-step nanofiber extraction and compounding technique, an integrated process was adopted to combine nanofibrillation and compounding into one step. Results showed that cotton fibers with a slight prefibrillation and hydrophobic surface modification were successfully fibrillated into tens to hundreds of nanometers in width during compounding. The nanofibers reinforced polypropylene composites exhibited significantly enhanced tensile and flexural strength and moduli. For instance, when 30% fibers from bleached white and indigo-dyed denim fabrics were introduced, the tensile moduli of the resultant composites reached 4.57 and 4.59 GPa, respectively, compared to 1.60 GPa, the modulus of neat PP. Meanwhile, denim fabrics had a remarkable reinforcing effect on the composites’ impact strength attributing to the hydrophobic indigo dyes that improved the interfacial bonding between cotton fibers and the matrix. The highest impact strength of denim reinforced composites was 4.96 kJ/m^2^ with 20% fiber loading; while the impact strength of neat polypropylene was 2.46 kJ/m^2^. The low water uptake of the composites further indicated the excellent adhesion at the filler/matrix interface. In general, a very promising processing technique to recycle cotton waste for high-value products was demonstrated.

## Introduction

The use of cellulosic fibers for reinforcing plastics has drawn growing interest in the industry due to the many advantageous properties of cellulose. Among these, high mechanical strength and stiffness, low density and thermal expansion, abundance in nature, low cost, and biodegradability are especially attractive for lightweight composites with enhanced recyclability and biodegradability^1,2^. Bast fibers and wood pulp have been studied more extensively than cotton as reinforcements. Although less researched, cotton is an attractive filler due to its high cellulose content and crystallinity, resulting in enhanced mechanical performance. Additionally, the abundance and low cost of post-consumer cotton waste make it more competitive than other cellulosic fiber^3–5^. The fact that more than 85% of cotton waste is landfilled or incinerated urges us to find new uses for this natural resource^6,7^.

The common route of producing cotton reinforced composites is to mix cotton in its original or shortened length into plastic for compounding and molding. The mechanical performance of the composites is limited because of the large fiber size and its hydrophilic nature, which result in poor interfacial bondings between fibers and the plastic. To improve mechanical properties, using nanocellulose (NC) has been studied and reported in recent years. NC is the elementary component of the plant cell’s hierarchical structure that confers its high mechanical strength^8^. NC possesses a high Young’s modulus of 100–130 GPa and a low density of approximately 1.5 g/cm^3^ compared to those of glass fibers at around 70 GPa and 2.6 g/cm^3^, respectively. Along with its low coefficient of thermal expansion (10^–7^/K) as well as abundance in nature, NC has attracted extensive interest as a reinforcing filler with the potential to replace carbon and glass fibers^9^.

There are two forms of NC, i.e., cellulose nanocrystals (CNCs) extracted from cellulosic fibers using strong acid hydrolysis and cellulose nanofibers (CNFs) obtained by mechanical grinding. CNCs have a size of approximately 3–5 nm in width and 50–500 nm in length with a high degree of crystallinity, while CNFs are 10–100 nm wide and 0.5– 5 μm long with more amorphous regions^10^. Although CNCs are stronger than CNFs, their reinforcing effect is not as good as that of CNFs, as reported by many studies. This is due to the higher aspect ratio of CNFs (140–500 vs. 15–40 for CNCs) and the nanofiber network formed, which enhances stress transfer within the composites^11–13^.

One of the challenges relating to commercially scaling up the NC reinforced composite manufacturing process is to achieve even dispersion of the hydrophilic NC in a hydrophobic plastic matrix and still retain its nano size without aggregation. In current composite processing, NC procuration is a prerequisite step^14,15^, where NCs are prepared and stored in an aqueous medium. Before melt-compounding with the plastic, NCs need to be dried. During the drying process, due to the large surface area and interfibrillar hydrogen bonds, NC tends to aggregate into particles of tens of microns, diminishing its reinforcing power. It was reported that directly mixing 5% of CNFs with poly(lactic acid) (PLA) resulted in composites with a similar Young’s modulus but 10% lower tensile strength than neat PLA due to CNF agglomeration^16^. Chemical modification of CNFs to replace some of its hydroxyl groups with hydrophobic ones by acetylation and alkenyl succinic anhydride modification^17–19^ can enhance the reinforcing effect.

This study investigated recycling cotton waste fabrics to produce lightweight polypropylene (PP) composites using an integrated nanofibrillation and compounding one-step process. Cotton waste fibers were mixed with PP for melt-compounding, during which both dispersion and fiber nanofibrillation occurred simultaneously, hence eliminating the separated CNF preparation and drying steps. A similar process was reported by Igarashi et al., who used wood pulp to reinforce high-density polyethylene and achieved excellent reinforcing results^18^. In the present study, cotton waste fabrics were pretreated mechanically to reduce the fiber size and chemically to modify the hydrophilicity. The effects of these pretreatments on facilitating the nanofibrillation process and improving fiber dispersion, along with the influence of fiber loading, were studied. We also compared the reinforcing effect of nanofibers from bleached white fabrics and blue denim fabrics dyed with hydrophobic indigo dyes. Various properties of the cotton/PP composites were evaluated, including morphology, tensile modulus and strength, flexural modulus and strength, impact resistance, water uptake, and surface contact angle.

## Material and method

### Material

Two types of fabrics, bleached white cotton and indigo-dyed blue denim, were kindly provided by Cotton Incorporated (USA). (2-Dodecen-1-yl) succinic anhydride (ASA) (Assay = 95%), *N*-methyl-2-pyrrolidone (NMP) (Assay = 99.5%), potassium carbonate (K_2_CO_3_), and calcium carbonate (CaCO_3_), and 4-dimethylaminopyridine (DMAP) were purchased from Sigma-Aldrich (USA). Maleic anhydride-grafted polypropylene (MAPP) pellets were from Honeywell International Incorporate (USA). Acetone, acetic acid (Assay = 99.7%), isopropanol (Assay = 99.5%), and ethanol (200 proof, anhydrous) were purchased from Fisher Scientific (USA). Polypropylene (Fortilene HB9200) was purchased from BP. Amoco Chemical Company (USA).

### Pretreatments of cotton fabrics

Fabrics were shredded into fibers with a Pallman Contra Selector Mill (Pallman, Germany). The shredded fibers were pre-fibrillated using a Super MassColloider (SMC) (MKC6-5J, Masuko Sangyo, Japan). Fibers suspended in water (2 wt.%) were fed to SMC for 20 passes at 25 °C. The SMC speed was 1500 RPM with a disk gap distance of 20 μm. SMC ground fibers were labeled as SMC-cotton.

In the ASA surface modification process, cotton fibers (40 g) were first dispersed in NMP (200 mL) directly or through solvent exchanged if in water. Then, a mixture of ASA (56 g), NMP (1.6 g), and K_2_CO_3_ (10 g) in NMP (20 mL) was added to the cotton NMP slurry and heated to 80 °C. The reaction was stopped after one hour, and fibers were washed in sequence with acetone, ethanol, aqueous acetic acid, distilled water, and isopropanol alcohol. The ASA-modified fibers were then collected and labeled as ASA-cotton.

### Fabrication of cotton/PP composites

Cotton fibers were mixed with MAPP powder, CaCO_3_, and one part PP powders in isopropanol. The mixture was blended in a food blender and dried overnight at 80 °C. The dried mixture was again blended in the food blender and dried further at 105 °C before extrusion. For cotton/PP compounding, the temperature of the extruder (Leistritz ZSE-18, Germany) was set at 175 °C with a screw speed of 200 RPM. Injection molded composite samples for mechanical testing were prepared using a MiniJet II piston molder (Sumitomo SE50D) at 170–190 °C zone and 50 °C model temperatures. All molded specimens were conditioned at 21 °C and 50% relative humidity for 7 days before further characterization.

### Determination of the degree of substitution

According to the literature^17,20^, the degree of substitution (DS) value of the ASA modified cellulose was calculated by titration. Specifically, 100 mg dried fiber samples were mixed with 40 mL of ethanol (75%) and heated to 50–60 °C for 30 min. Then, 40 mL of 0.5 mol/L NaOH solution was added and heated at 50 °C for 15 min, followed by stirring at room temperature for 12 h. The mixture was titrated with 0.1 mol/L HCl solution. The DS was calculated from the following equation:

1 $$DS=\frac{A\times 162}{1-266\times A}$$

where 162 is the molar mass of cellulose per unit (162 g/mol), 266 is the molar mass of ASA (266 g/mol), *A* is the molar concentration of ASA (mol/g) in the dry fiber samples calculated from the following equation:

2 $$A= \frac{{c}_{HCl}\left({V-V}_{m}\right)}{{W}_{s}}$$

where *V* is the volume of HCl (mL) used for the titration of the neat fiber, *V*_*m*_ is the volume of HCl (mL) used for titration of the ASA modified fibers, ${c}_{HCl}$ is the HCl concentration (mol/mL), and *W*_s_ is the total weight (g) of the dried fiber sample. The procedures were repeated three times and the avergage value of DS were obtained.

### Fourier-transform infrared (FTIR) analysis

Infrared spectra of cotton fibers were measured using Fourier-Transform Infrared Spectroscopy (Nicolet iS-50, Thermal Fisher Scientific, USA) with a resolution of 4 cm^−1^ and 64 scans under the attenuated total reflectance (ATR) mode. The spectra were normalized using a 1315 cm^−1^ peak of the typical –CH_2_ vibration of cellulose.

### Scanning electron microscopy (SEM)

The morphology of cotton fibers and cotton/PP composites were examined using Field Emission Scanning Electron Microscopy (FE SEM, Quanta 200F, USA). All samples were sputter-coated with 20 nm gold before characterization. The electron field was 30 kV.

### Mechanical testing of cotton/PP composites

The tensile properties were evaluated using an Instron material tester (Model 4466, Instron, USA), following the ASTM D638 Standard Test Method for Tensile Properties of Plastics. The crosshead speed was 5 mm/min, and the grip length was 45 mm. The flexural properties were also evaluated using the Instron tester following ASTM D790 Standard Test Methods for Flexural Properties of Unreinforced and Reinforced Plastics and Electrical Insulating Materials. The deflection speed was 14 mm/min (strain rate = 0.01 mm/min). The notched impact strength was determined by a Basic Pendulum Impact tester (BPI-0-1, Dynisco, USA) following ASTM D256 Standard Test Methods for Determining the Izod Pendulum Impact Resistance of Plastics. All testing results were calculated based on at least five replications.

### Rheological property of cotton/PP composites

The melt rheology of cotton/PP composites was evaluated by a rheometer (Discovery Hybrid HR-2, TA instruments, USA) configured with the parallel plates (25 mm diameter) in 1 mm spacing. The angular frequency varied from 0.01 to 500 rad/s, and the temperature was 180 °C. The sweeping strain (0.5%) at the linear viscoelastic region was determined by dynamic strain sweep.

### Water absorption of cotton/PP composites

The water absorption test was conducted to evaluate the water absorption value according to ASTM D570 Standard Test Method for Water Absorption of Plastics. The composites were dried overnight at 105 °C and then immersed in distilled water for 24, 48, 96, 144, 196, 288, and 432 h at room temperature. Specimens retrieved from water were wiped dry and weighed using an electronic balance. The percentage increase in weight was calculated. Three samples were measured for each time interval.

### Contact angle of cotton/PP composites

The water contact angles of the surface of cotton/PP composites were measured with a Video Contact Angle System (VCA Optima, AST products Co., USA). The contact angles were recorded at room temperature after the water droplet immediately contacted the specimen surface. Each measurement was repeated three times.

## Results and discussion

### Characteristics of pretreated cotton waste fabrics

**Figure 1:**
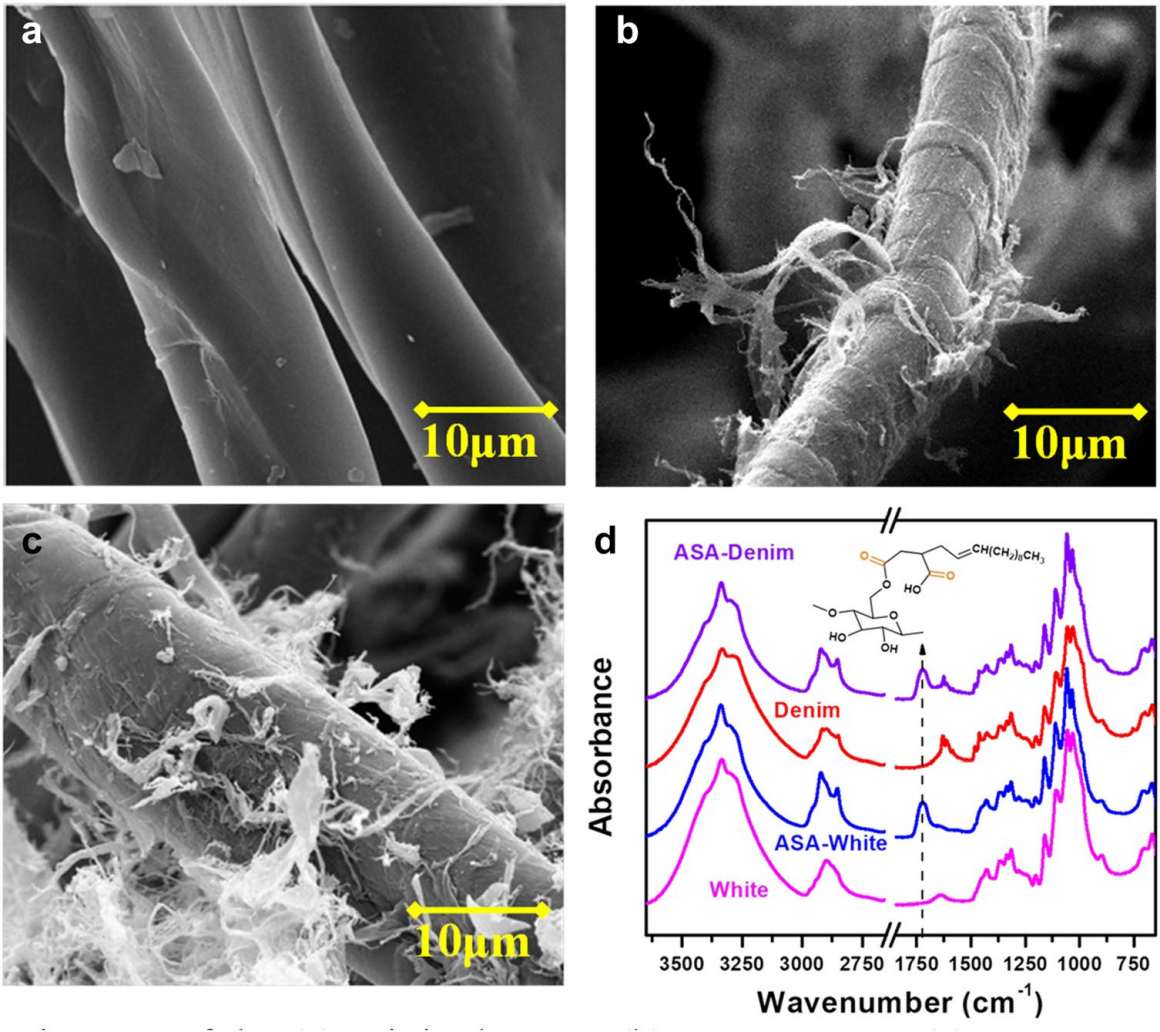
SEM images of the (**a**) original cotton, (**b**) SMC-cotton, (**c**) ASA-SMC-cotton, and (**d**) FTIR spectra of the original cotton and ASA-SMC-cotton for both bleached white fibers and denim fibers. (The increased intensity at ~ 1740 cm^−1^ due to the carbonyl groups after ASA modification was indicated by the arrow).

To facilitate fiber fibrillation and the compounding process, cotton fabrics were shredded into short fibers of approximately 2 mm with a Contra Selector Mill followed by SMC pre-fibrillation. SEM images in Fig. 1a–c show the morphologies of the original cotton fibers shredded from the bleached white fabric, SMC ground white cotton (SMC-cotton) after 20 passes, and ASA modified white SMC-cotton (ASA-SMC-cotton). The shredded fibers exhibited the features of virgin cotton, i.e., smooth surface with ribbon-like convolutions. The purpose of SMC grinding was to loosen the fiber structure to facilitate the following chemical modification and nanofibrillation. Since cotton fibers have a compact structure and high crystallinity compared to other natural cellulosic fibers, 20 passes of grinding only affected the fiber surface and brought some fibrils out onto the surface. The resultant rougher fiber surface, protruding fibrils, and increased surface area were expected to enhance fibers’ adhesion to the PP matrix in the composites^17^. ASA-SMC-cotton had a similar morphology to SMC-cotton but with more fibrils on the surface due to the agitation during ASA modification in the aqueous reaction environment. No difference was observed in morphologies between cotton fibers from the bleached white and indigo-dyed blue fabrics.

FTIR was used to inspect the grafting of ASA onto cotton fibers, including white and denim (Fig. 1d). A new band appeared at around 1740 cm^−1^ on the FTIR spectra of the modified fibers. This was ascribed to the carbonyl bonds of esters and carboxylic acids of ASA after the ring opening of anhydrides, confirming that ASA was successfully grafted. The intensity of the bands at around 2900 cm^−1^ was also increased, corresponding to the C–H stretching vibration of CH_2_ and CH_3_ groups from the aliphatic chain of ASA. Through calculating the peak height in the FTIR spectra and using chemical titration, the degree of substitution (DS) of ASA-SMC-cotton was between 0.23 and 0.25 for both white and denim fibers. From this relatively small DS value, it is reasonable to deduce that only the hydroxyl groups of cellulose molecules at the fiber surface were esterified, while the bulk properties of fibers remained unchanged.

### Cotton/PP composite preparation and characterization

**Figure 2:**
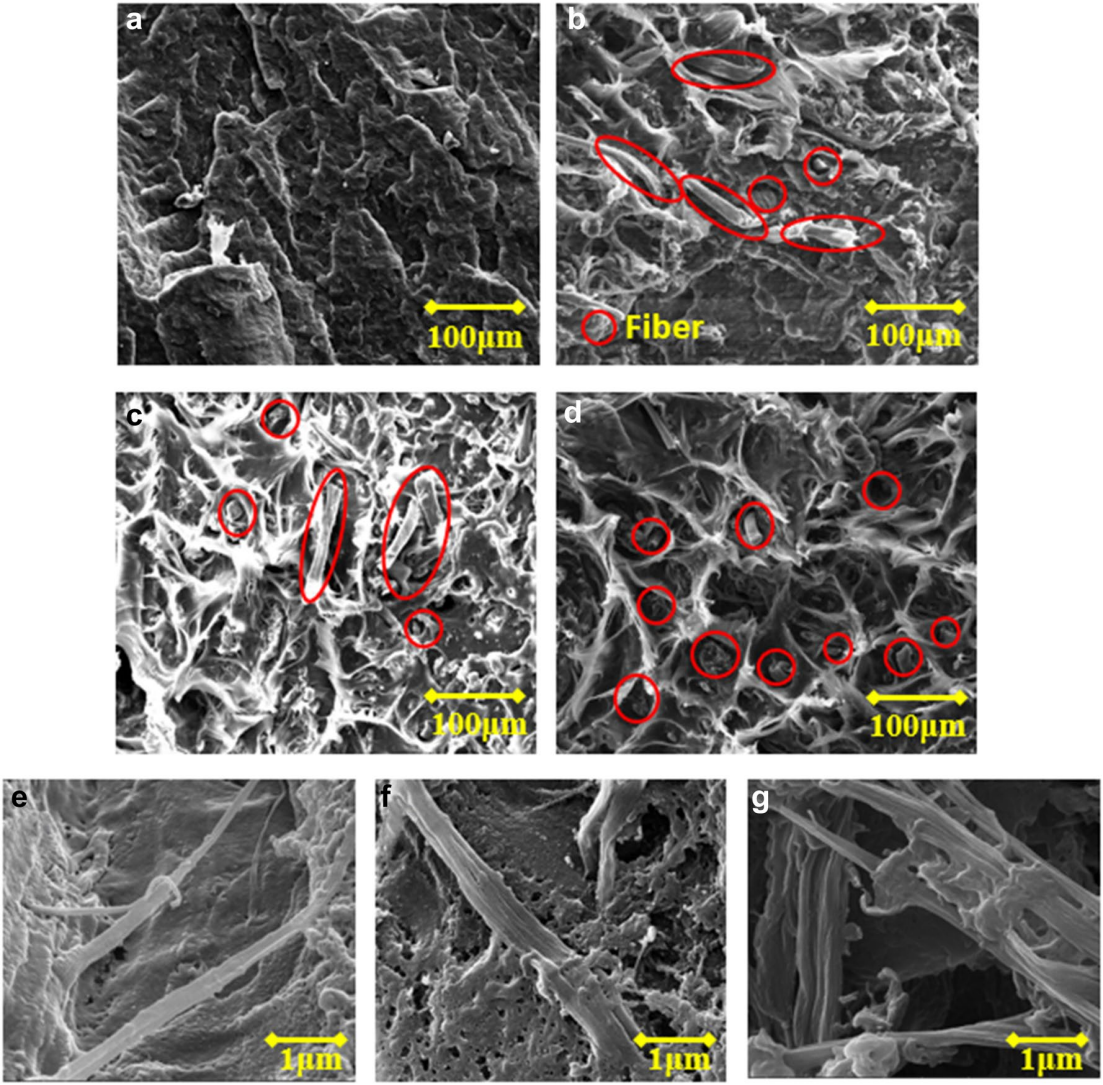
SEM images of tensile fractures of (**a**) neat PP, (**b**) raw cotton/PP, (**c**) ASA-SMC-cotton/PP with one time compounding, (**d**) ASA-SMC-cotton/PP with double compounding, and (**e**–**g**) cellulose nanofibers in the ASA-SMC-cotton/PP double compounded composites. Broken fibers at the fracture surfaces are circled in (**b**–**d**).

The different water affinities of cellulose and polypropylene pose a challenge for homogeneous dispersion of cotton fibers in the PP matrix, even after ASA modifications as reported^21^. In this study, cotton fibers were pre-mixed with some PP powders, the compatibilizer MAPP, and CaCO_3_ via blending to obtain a cotton/PP mixture. The mixture was fed into the extruder with the rest of PP powders for compounding. Pellets obtained after compounding were processed through the extruder for a second time for improved composite uniformity. Figure 2a–d show the tensile fractures of neat PP, raw cotton/PP, ASA-SMC-cotton/PP with single compounding, and ASA-SMC-cotton/PP with double compounding. Neat PP had a smooth fracture surface. The raw cotton/PP composites had a less smooth fracture surface than the neat PP. Fiber pullout from the matrix was clearly seen in addition to voids between cotton fibers and the PP matrix. This suggested that the interfacial bonding was not strong enough to sustain the deformation stress, leading to debonding and fiber pullout. In comparison, ASA-SMC-cotton/PP had a much rougher fracturing surface showing ductile stretching of PP molecular chains that were surrounding cotton fibers. The morphology indicated that the deformation stress was effectively transferred from the PP matrix to cotton fibers during testing due to the strong interfacial bonding. Hence, much less fiber pullout was observed. Further improved interfacial bonding between the PP matrix and cotton fibers from single compounding to double compounding was evidenced by the SEM images. Therefore, a double compounding process was adopted to prepare the cotton/PP composites presented in the following sections.

The morphologies of cotton fibers after compounding were examined via SEM images of composites that were etched in xylene for 3 s. Part of the PP matrix was removed by xylene and fibers inside the composites were exposed. As shown in Fig. 2e–g, these fibers had submicron diameters, approximately 100–500 nm, and they formed fibril networks. The size of fibers before compounding was generally more than 10 μm. The kneading motion during compounding splitted fibers into nanofibrils and dispersed them in the PP matrix simultaneously. The smaller fiber size, interconnected fibril network, and improved interfacial adhesion were all positive factors promoting the fiber reinforcing outcomes as evidenced by the composite mechanical properties to be discussed below.

### Mechanical properties of cotton/PP composites

#### Tensile properties

**Figure 3:**
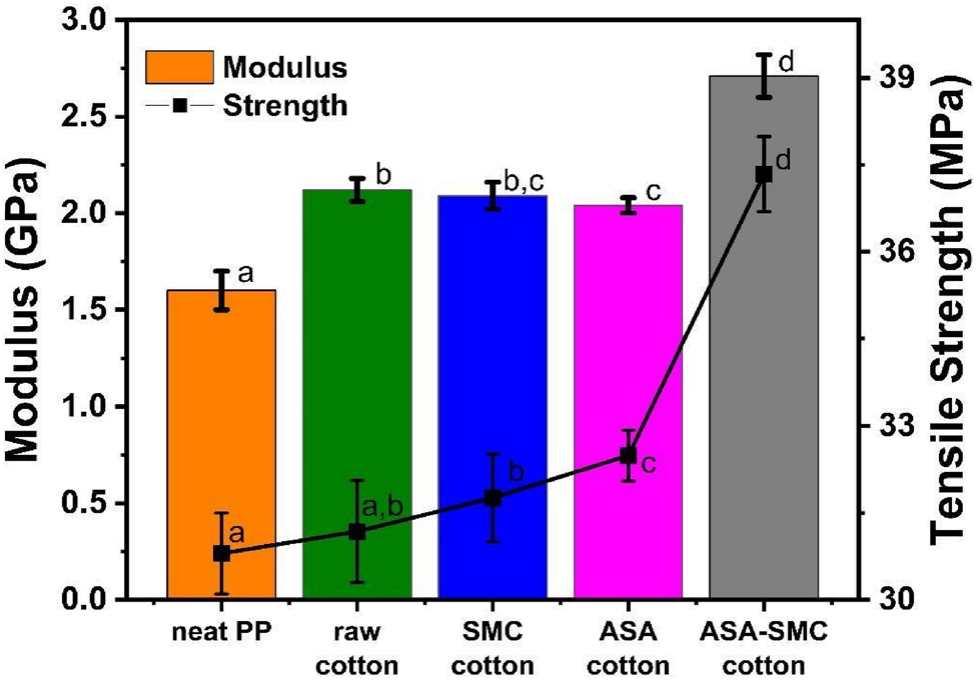
Tensile strength and modulus of neat PP and PP composites reinforced with 10% white cotton (raw cotton, SMC-cotton, ASA-cotton, and ASA-SMC-cotton). Different letters (a-d) indicate significant differences (p < 0.05) using Student’s t-test for both strength and modulus.

Tensile strength, modulus, and elongation of the cotton/PP composites were evaluated. Since fiber size, aspect ratio, fiber loading, and interfacial bonding between fillers and the plastic matrix are among the important factors that affect the tensile properties of fiber reinforced composites^22^, we studied the influence of fiber pretreatments, cotton fiber loading, and the type of cotton fabrics in this project. Figure 3 displays the tensile strength and modulus of neat PP and white cotton/PP composites reinforced with 10% raw cotton, 10% SMC-cotton, 10% ASA-cotton, and 10% ASA-SMC-cotton. Given the same cotton fiber loading, the average tensile strength of the cotton/PP composites increased in the order of neat PP, raw cotton/PP, SMC-cotton/PP, ASA-cotton/PP, and ASA-SMC-cotton/PP. Student’s t-test analysis revealed that all treatments (SMC, ASA, and both) significantly increased tensile strength compared to neat PP at a 5% significance level. The differences between neat PP (30.80 MPa) and raw cotton/PP (31.18 MPa) were not significant. This is in line with what has been reported in the literature^23–29^. Due to their different polarities, hydrophilic cotton fibers without a pretreatment could not form strong interfacial bonding with hydrophobic PP matrix, leading to poor reinforcing effect. This is evidenced by the pullout phenomenon in the SEM images of the fractures, where deboned fibers with a bare surface were observed. The raw cotton/PP and SMC-cotton/PP (31.76 MPa) had comparable strength. ASA-SMC-cotton/PP showed noticeably higher tensile strength (37.34 MPa) than ASA-cotton/PP (32.49 MPa) and SMC-cotton/PP, demonstrating the effect of combining the ASA and SMC treatments. The high strength of ASA-SMC-cotton/PP was consistent with the tensile fractured surface showing less fiber pullout and voids between fibers and the PP matrix. The grafted hydrophobic ASA on fiber surfaces enhanced the interfacial bonding between cotton fibers and the PP matrix.

The moduli of all these cotton reinforced composites were enhanced from 1.60 GPa for neat PP to 2.12, 2.09, 2.04, and 2.71 GPa for raw cotton/PP, SMC-cotton/PP, ASA-cotton/PP, and ASA-SMC-cotton/PP, respectively. Similar to the tensile strength, ASA-SMC-cotton had the best reinforcement in modulus as well.

**Figure 4:**
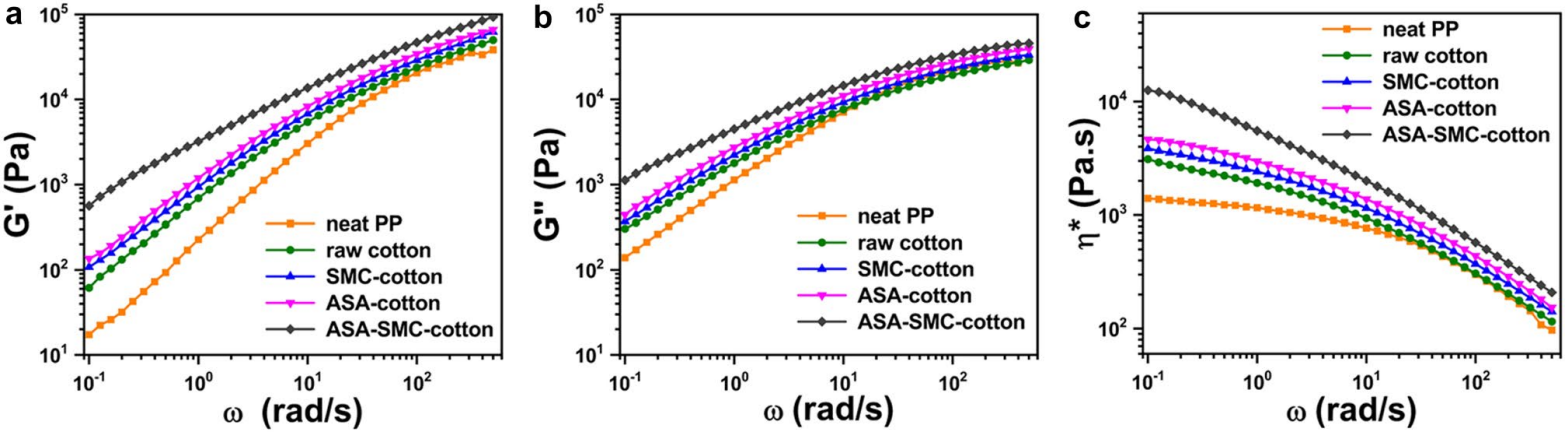
Plots of (**a**) storage modulus, (**b**) loss modulus, and (**c**) complex viscosity versus angular frequency for PP composites reinforced with 10% white raw cotton, SMC-cotton, ASA-cotton, and ASA-SMC-cotton.

The better reinforcing effect of ASA-SMC-cotton was evidenced by not only the tensile fractures, the highest tensile strength and modulus, but also their melt rheology shown in Fig. 4. ASA-SMC-cotton had the highest storage modulus (G′), loss modulus (G″), and complex viscosity (η*) due to the strong interfacial interaction between cotton fibers and the PP matrix^30–34^. The well fibrillated CNFs and their interconnected network (as shown in Fig. 2e–g) caused more internal frictions than other composites. When the shear rate was low (e.g., ω < 1 rad/s), the high-stiff and networked CNFs were “frozen” in the PP matrix, presenting high η*; increasing shear rate triggered more shear thinning behavior, leading to a better flowability. The 10% ASA-SMC-cotton/PP composites exhibited decent melt processability comparable to that of neat PP, indicating that it was feasible to further increase fiber loading in the composites.

The effect of the fiber loading on tensile properties of both white cotton and denim cotton was studied by mixing 5, 10, 20, and 30% of ASA-SMC-cotton with PP. Table 1 contains data for the strength, Young’s modulus, and elongation of the composites. Increasing fiber loading clearly enhanced both tensile strength and modulus. The percent increase of strength for white cotton/PP composites approximately doubled the cotton loading at 10, 21, 46, and 59% for 5, 10, 20, and 30% fiber loadings, respectively. As expected, the moduli of cotton/PP composites were much higher than that of neat PP. A similar trend was observed for denim cotton composites, which indicated that the indigo dyes did not affect the cotton reinforcing effect in terms of tensile properties. The fact that increasing fiber loading enhanced tensile strength further signified the reinforcing effect of combining ASA treatment and prefilbrilation by improving the interfacial bonding. Generally, it is challenging to increase the tensile strength of hydrophobic plastics by adding high content of natural fibers without treatments.

**Table 1:** Tensile properties of cotton/PP composites with different cotton loadings.

Cotton content	Tensile strength (MPa)	Strength increase compared to PP (%)	Tensile modulus (GPa)	Modulus increase compared to PP (%)	Elongation at break (%)
Neat PP	30.80 ± 0.70^a^	0	1.60 ± 0.10^a^	0	> 100
White cotton 5%	33.95 ± 0.53^b^	10.2	2.30 ± 0.11^b^	43.8	17.14 ± 0.49
White cotton 10%	37.34 ± 0.65^c^	21.2	2.71 ± 0.11^d^	69.4	10.6 ± 0.95
White cotton 20%	45.05 ± 1.00^d^	46.3	3.78 ± 0.09^d^	136.3	6.22 ± 0.67
White cotton 30%	48.97 ± 1.11^e^	59.0	4.57 ± 0.12^e^	185.6	3.74 ± 0.60
Denim cotton 5%	34.43 ± 0.36^b^	11.2	2.15 ± 0.04^f^	34.3	12.71 ± 1.57
Denim cotton 10%	37.77 ± 0.36^f^	22.6	2.50 ± 0.05^g^	56.2	8.68 ± 0.78
Denim cotton 20%	45.24 ± 0.44^d^	46.7	3.36 ± 0.08^h^	110.0	6.25 ± 0.21
Denim cotton 30%	51.50 ± 0.97^g^	67.2	4.59 ± 0.09^i^	186.9	4.16 ± 0.50

Different letters indicate significant differences (p < 0.05) using Students’ t-test for strength and modulus.

#### Flexural properties

**Figure 5:**
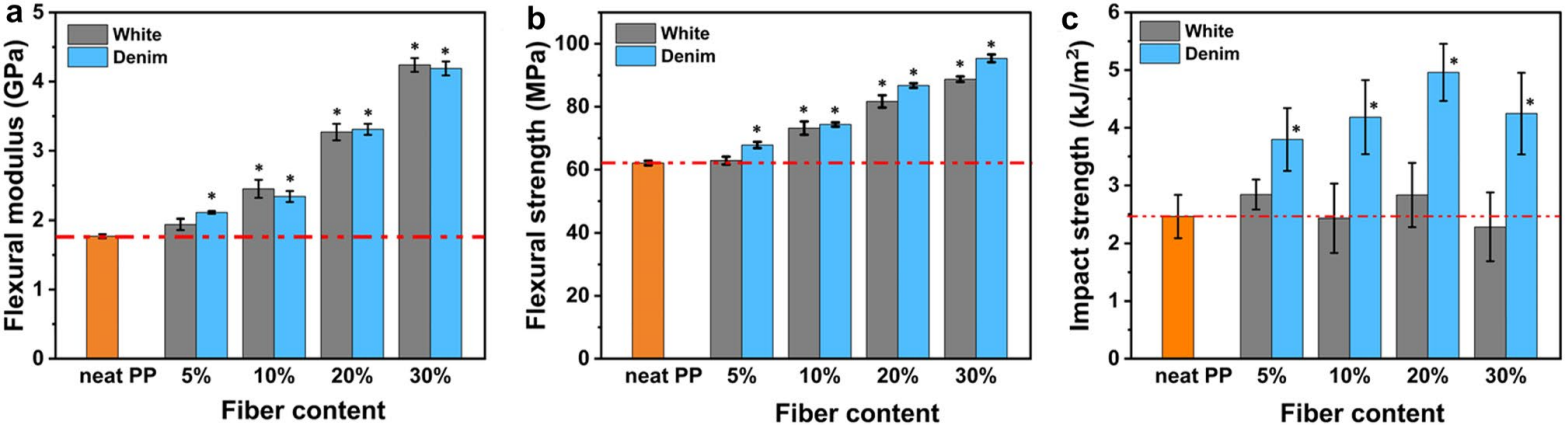
Graphs for the (**a**) flexural modulus, (**b**) flexural strength, and (**c**) impact strength of neat PP and ASA-SMC-cotton/PP composites with different fiber loadings. The star indicates the difference with neat PP was significant (p < 0.05) using Student’s t-test.

Figure 5a,b present the flexural bending properties of white cotton and denim cotton reinforced composites with different fiber loadings. The neat PP samples had flexural modulus and bending strength of 1.77 GPa and 62.11 MPa, respectively. Adding cotton fibers from 5 to 30% improved both properties. With 5, 10, 20, and 30% white cotton, the composites had a flexural strength of 62.89, 73.19, 81.70, and 88.76 MPa, respectively, reflecting a 0.8, 17.3, 31.0, and 42.3% increase from neat PP. The bending strength of denim/PP composites had an increase of 9.0, 19.0, 40.0, and 53.6% from neat PP with denim cotton fiber loadings from 5 to 30%. The enhancement of the flexural modulus of cotton/PP was more substantial than the flexural strength. When 30% cotton was added, moduli of white cotton and denim cotton composites increased 140.5% and 137.8%, respectively. CNF had good reinforcing capabilities because the nanofibers were highly rigid with Young’s modulus as high as 138 GPa, and they hardly deformed appreciably under outside load^34,35^.

The results indicated that the cotton/PP composites had effective stress transfer under bending load. There were two factors that contributed to this. First, fibers were dispersed evenly in the composites. Second, the fibrillation during kneading created nanofiber networks that facilitated load transfer among fibers to reduce stress concentration. With higher cotton content, fibers were closer and had a larger chance of having contact with each other to form a “percolation effect”^36,37^. Overall, both 30% white and 30% denim showed great reinforcement outcomes as their modulus and strength were significantly increased. These results are very promising compared to some CNF/PP composites reported in the literature. For instance, in Wang’s study^38^, adding 30% spray-dried CNF in PP enhanced the flexural modulus of PP from 1.7 to 2.4 GPa; while the improvement in tensile strength was neglectable, from 32 MPa of the neat PP to 33 MPa of the composites.

#### Impact resistance

**Figure 6:**
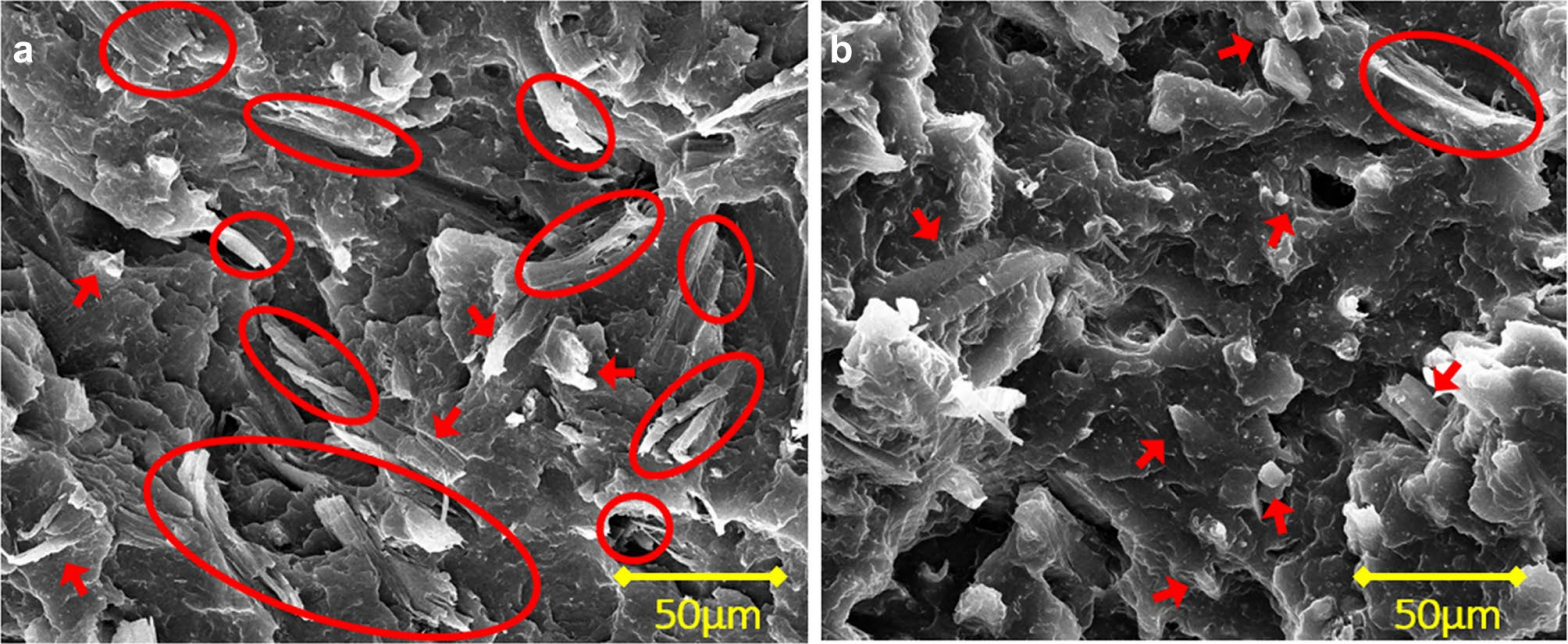
Impact fracture of 20% ASA-SMC denim cotton (**a**) and white cotton (**b**) composites. Peeled fibers at the fracture surface are circled and arrows point to broken fibers.

Tensile and flexural strength evaluate the effect of slowly exerted deformation on the material, whereas the impact strength measures the capability to resist a sudden force that simulates many real application scenarios. Some studies reported reduced impact strength when CNF was added to a plastic matrix^39,40^. In this study, for the white cotton composites, the average impact resistance of 5% and 20% ASA-SMC-cotton/PP were both at 2.84 kJ/m^2^, larger than that of neat PP, which was 2.46 kJ/m^2^. The values for 10% and 30% were lower at 2.43 kJ/m^2^ and 2.28 kJ/m^2^, respectively, as shown in Fig. 5c. However, none of the differences was statistically significant at a 5% significance level, meaning that adding white cotton fibers at different loadings did not affect the impact resistance of PP. Comparatively, the denim cotton had a very different effect at all loadings. The impact resistance was at 3.79, 4.18, 4.96, and 4.24 kJ/m^2^, with fiber loadings increased from 5 to 30%. These values were significantly higher than the neat PP and white cotton/PP at the same loading. Since interfacial adhesion has a more important influence on impact strength than other mechanical properties^38,41^, the results suggested that the denim cotton might have better interfacial adhesion with the PP matrix than white cotton due to the hydrophobic indigo dyes, which might also have facilitated nanofibrillation during compounding to reduce fiber size and improved composite uniformity. This is difficult to verify directly using experimental techniques. However, when examining the impact fracture, more fiber peeling was observed on the denim cotton/PP composites than on the white cotton composites, as shown in Fig. 6. Fibers showed very good adhesion with the PP matrix at the fracture surface. More fiber pullouts and debonding were observed on the white cotton/PP fracture surface.

### Water absorption and surface contact angle

**Figure 7:**
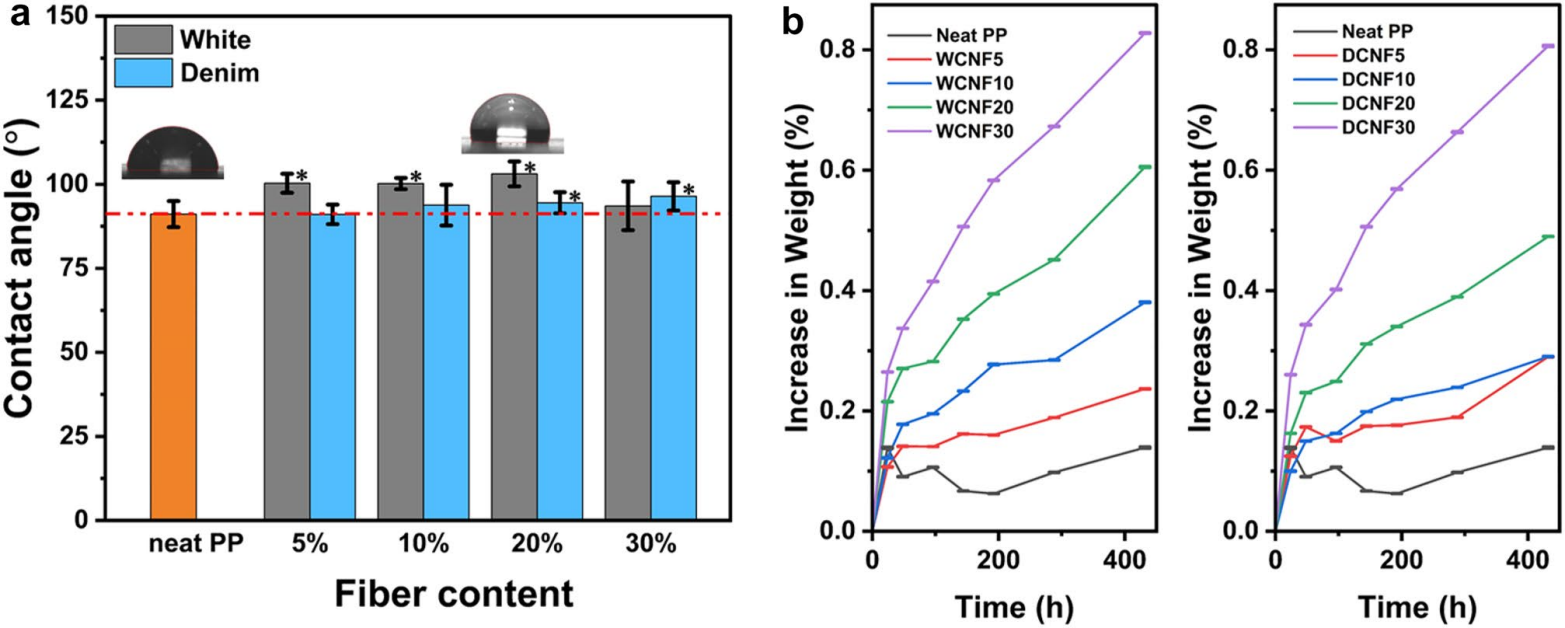
Surface contact angles (**a**) and water uptake (**b**) of neat PP and ASA-SMC-cotton/PP composite. The star indicates the difference with neat PP was significant (p < 0.05) using Student’s t-test.

The moisture resistance of natural fiber reinforced composites is important as hydrophilic fibers absorb moisture, which can degrade the interfacial bonding between fibers and the matrix, resulting in deteriorated mechanical, thermal, and physical properties of the composite and affecting their end uses^42^. Figure 7a depicts the contact angles of the cotton/PP composites. The figure demonstrates that the inclusion of cotton fibers did not increase the surface wettability of the composites. The contact angles of white cotton/PP composites were larger than that of neat PP, except for the one with 30% cotton. For denim, higher cotton loadings at 20% and 30% significantly enhanced the contact angle. The increase in water contact angle may be attributable to the ASA modification, which improved the adhesion between cotton fibers and PP and also reduced the surface polarity of the composites’ surface^43–46^. Water uptake is another measure of moisture resistance. Figure 7b shows the weight increase of the cotton/PP composites soaked in water for up to 18 days. Higher fiber loading and lengthened soaking time resulted in increased water absorption. The largest water uptake among all the composites was only 0.83% after 432 h. The value was much lower than those reported in the literature. For example, the water absorption of PP composites containing 30% of wood flour and cellulose from pulp reached 1.3% and 6%, respectively, as reported by Ichazo^47^ and Espert^48^. In Abebayehu’s research^49^, sisal fiber-reinforced PP composites with 10% fiber had an absorption of 6.5% after 144 h. This study’s low water absorption results further suggested excellent cotton and PP interfacial bonding. A protection layer of PP adhering to the fiber surfaces impeded moisture penetration and absorption by the hydrophilic cotton fibers.

## Conclusion

In conclusion, nanocellulose reinforced lightweight composites were successfully manufactured using an integrated nanofibrillation-compounding process from both bleached white and indigo-dyed denim fabrics. It was found that the pre-fibrillation of cotton followed by ASA modification greatly facilitated the nanofibrillation and improved the dispersion of cotton fibers in the PP matrix. Given the same fiber loading, the average strength of the composites increased in the order of neat PP, raw cotton/PP, SMC-cotton/PP, ASA-cotton/PP, and ASA-SMC-cotton/PP. Fiber grinding and ASA chemical modification combined had the most reinforcing effect. As cotton content increased, the strength and modulus of both tensile testing and bending testing increased. There was no marked difference in the tensile and flexural properties between composites reinforced with bleached white cotton and indigo-dyed denim cotton. However, denim cotton had a great advantage over white cotton in producing better nanocellulose reinforced composites regarding impact resistance. Adding bleached white cotton to PP at loadings from 5 to 30% did not affect the impact resistance, while denim cotton significantly enhanced the impact resistance of the composite. This was very likely due to the intrinsic hydrophobic nature of indigo dyes on the fibril surfaces that increased the interfacial bonding between cotton and PP. The obtained nanocellulose reinforced composites had very low water absorption, indicating very good matrix/fibers interfacial bondings between the modified cotton fibers and the PP matrix. This study provided a promising route for applying cotton wastes in the nanocellulose reinforced composites using an integrated nanofibrillation-compounding process. The nanocellulose reinforced composites are expected to find applications in the transportation industry, such as interior components of automobiles and aircraft (e.g., seats, handles, storage compartments and bins, ceiling).

## Data Availability

The datasets generated during and/or analyzed during the current study are available from the corresponding author upon reasonable request.
